# Biomarkers as therapy monitoring for postmenopausal osteoporosis: a systematic review

**DOI:** 10.1186/s13018-021-02474-7

**Published:** 2021-05-18

**Authors:** Filippo Migliorini, Nicola Maffulli, Filippo Spiezia, Markus Tingart, Peretti Giuseppe Maria, Giorgino Riccardo

**Affiliations:** 1 Department of Orthopaedics, University Clinic Aachen, RWTH Aachen University Clinic, Pauwelsstraße 30, 52074 Aachen, Germany; 2 Department of Medicine, Surgery and Dentistry, University of Salerno, Via S. Allende, 84081 Baronissi, SA Italy; 3 School of Pharmacy and Bioengineering, Keele University Faculty of Medicine, Thornburrow Drive, Stoke on Trent, England; 4 Centre for Sports and Exercise Medicine, Mile End Hospital, Barts and the London School of Medicine and Dentistry, Queen Mary University of London, 275 Bancroft Road, London, E1 4DG England; 5 Department of Orthopedics and Trauma Surgery, Ospedale San Carlo Potenza, Via Potito Petrone, Potenza, Italy; 6 Department of Biomedical Sciences for Health, University of Milan, Milan, Italy; 7 Orthopedics and Traumatology, University of Milan, IRCCS Istituto Ortopedico Galeazzi, Milan, Italy

**Keywords:** Osteoporosis, Postmenopausal, Biomarkers, Therapy monitoring

## Abstract

**Background:**

Biochemical markers of bone turnover (BTMs), such as bone alkaline phosphatase (bALP), procollagen type I N propeptide (PINP), serum cross-linked C-telopeptides of type I collagen (bCTx), and urinary cross-linked N-telopeptides of type I collagen (NTx), are commonly used for therapy monitoring purposes for osteoporotic patients. The present study evaluated the potential role of BTMs as therapy monitoring.

**Methods:**

All randomized clinical trials (RCTs) comparing two or more pharmacological treatments for postmenopausal osteoporosis were accessed. Only studies that reported the value of bALP, PINP, bCTx, and NTx at last follow-up were included. A multivariate analysis was performed to assess associations between these biomarkers and clinical outcomes and rate of adverse events in patients with postmenopausal osteoporosis. A multiple linear model regression analysis through the Pearson product-moment correlation coefficient was used.

**Results:**

A total of 16 RCTs (14,446 patients) were included. The median age was 67 years, and the median BMI 25.4 kg/m^2^. The median vertebral BMD was 0.82, hip BMD 0.79, and femur BMD 0.64 g/cm^2^. The ANOVA test found optimal within-group variance concerning mean age, body mass index, and BMD. Greater bALP was associated with lower femoral BMD (*P* = 0.01). Greater NTx was associated with a greater number of non-vertebral fractures (*P* = 0.02). Greater NTx was associated with greater rate of therapy discontinuation (*P* = 0.04). No other statistically significant associations were detected.

**Conclusion:**

Our analysis supports the adoption of BTMs in therapy monitoring of osteoporotic patients.

**Level of evidence:**

Level I, systematic review of RCTs.

## Introduction

Bone is highly dynamic with resorption and ossification to maintain tissue homeostasis [1, 2]. Bone alkaline phosphatase (bALP) and procollagen type I N propeptide (PINP) have been considered biomarkers of bone ossification, while serum cross-linked C-telopeptides of type I collagen (bCTx) and urinary cross-linked N-telopeptides of type I collagen (NTx) are indicators of bone resorption [3–8]. Bone turnover markers (BTMs) highlight the dynamic balance of the bone tissue [4, 9]. Markers of ossification (bALP and PINP) derive from the procollagen metabolism or from osteoblasts. Markers of resorption (bCTx and NTx) are produced by osteoclasts or result from collagen degradation processes [2, 4]. BTMs are influenced by several endogenous factors, such as gender, age, ethnicity, fracture, and associated diseases [10–13]. Exogenous factors, such as circadian rhythm, seasonal variation, diet, and exercise, also influence BTMs [14–17]. Recently, many studies used BTMs to monitor the efficacy and safety of drugs influencing bone turnover [18–23] and as therapy monitors in postmenopausal osteoporosis [24–26]. Although the use of these biomarkers in clinical practice is common, their role as therapy monitors is still unclear [27, 28]. Indeed, no previous studies performed a systematical evaluation of their potential as therapy monitors in postmenopausal osteoporosis.

The purpose of the study was to explore the potential of bALP, PINP, bCTx, and NTx in therapy monitoring for postmenopausal osteoporosis, investigating their association with bone mineral density (BMD) and the rate of adverse events.

## Material and methods

### Search strategy

This study was conducted according to the Preferred Reporting Items for Systematic Reviews and Meta-Analyses: the PRISMA guidelines [29]. The PICOTD algorithm was preliminarily set out:

- P (Problem): Postmenopausal osteoporosis
- I (Intervention): Therapy monitoring
- C (Comparison): bALP, PINP, bCTx, NTx
- O (Outcomes): BMD, rate of fractures, and adverse events
- T (Timing): Minimum 6 months of follow-up
- D (Design): RCTs

### Literature search

Two independent authors (**;**) performed the literature search in April 2021. The following databases were accessed: PubMed, Google Scholar, EMBASE, and Scopus. No time constrains were used for the search. The following keywords were used in combination: *osteoporosis*, *treatment*, *management*, *drug*, *pharmacology*, *pharmacological*, *medicament*, *mineral*, *density*, *bone*, *BMD*, *bone alkaline phosphatase*, *ALP*, *procollagen type I N propeptide*, *PINP*, *serum cross-linked C-telopeptides of type I collagen*, *CTx*, *urinary cross-linked N-telopeptides of type I collagen*, *NTx*, *premenopausal*, *spine*, *pathological*, *fragility*, *fractures*, *hip*, *vertebral*, *disability*, *adverse events*, *Calcium*, *Vitamin D*, *PTH*, *osteoblast*, and *osteoclast*. The same authors independently performed the initial screening. If the title and abstract matched the topic, the article full-text was accessed. A cross reference of the bibliographies was also performed to identify further studies.

### Eligibility criteria

All randomized clinical trials (RCTs) comparing two or more pharmacological treatments for postmenopausal osteoporosis were accessed. According to the authors’ language capabilities, articles in English, French, German, Italian, Portuguese, and Spanish were eligible. Only level I studies, according to Oxford Centre of Evidence-Based Medicine [30], were considered for inclusion. Only articles reporting quantitative data under the outcomes of interest were eligible. Only clinical studies that reported the amount of bALP, PINP, bCTx, and NTx at last follow-up were included. Articles including patients with secondary osteoporosis were excluded. Studies concerning patients with tumors and/or bone metastases were also not included. Studies reporting data on patients with iatrogenic-induced menopausal and those on pediatric and/ or adolescent patients were not included. Combined therapies with multiple drugs were also not considered in the present study. Studies regarding selected patients undergoing immunosuppressive therapies or organ transplantation were also not considered. Studies with follow-up shorter than 6 months were not eligible, nor where those involving less than 10 patients. Studies reporting data of combined therapy with multiple anti-osteoporotic drugs were also not included. Missing data under these endpoints warranted the exclusion from the present work.

### Data extraction and outcomes of interests

Two authors (**;**) independently performed data extraction. Study generalities (author, year, journal, duration of the follow-up) and patient baseline demographic information were collected: number of samples and related mean age, mean body mass index (BMI), and mean bone mass index (BMD) of the spine, hip, and femur neck. Data concerning the following endpoints were collected at last follow-up: rate of vertebral, femoral, and hip osteoporotic fractures. Further, data concerning the following complications were collected: serious adverse events and those leading to study discontinuation, gastrointestinal events, musculoskeletal events, and mortality. Data concerning bALP, PINP, bCTx, and NTx were extracted at last follow-up. The ultimate aim was to assess association between biomarkers and clinical outcomes at last follow-up in terms of BMD, rate of pathological fractures, and adverse events.

### Methodological quality assessment

The methodological quality assessment was made through the risk of bias summary tool of the Review Manager Software (The Nordic Cochrane Collaboration, Copenhagen). The following risks of bias were evaluated: selection, detection, performance, reporting, attrition, and other sources of bias.

### Statistical analysis

The statistical analyses were performed by the main author (**). The IBM SPSS software version 25 was used to assess baseline data. The Shapiro-Wilk test was performed to investigate data distribution. For normal data, mean and standard deviation (SD) were calculated. For non-parametric data, median and interquartile range (IQR) were calculated. The Student *T*-test was used to assess significance for parametric data, while the Mann-Whitney *U*-test was used for non-parametric variables. Values of *P* < 0.05 considered statistically significant. A multivariate analysis was performed to assess associations between biomarkers and clinical outcomes at last follow-up. The STATA Software/MP (StataCorporation, College Station, TX, USA) was used for the statistical analyses. A multiple linear model regression analysis through the Pearson product-moment correlation coefficient (*r*) was used. The Cauchy–Schwarz formula was used for inequality: +1 was considered as positive linear correlation, and −1 a negative one. Values of 0.1< | *r* | < 0.3, 0.3< | *r* | < 0.5, and | *r* | > 0.5 were considered to have weak, moderate, and strong correlation, respectively. The overall significance was performed through the *χ*^2^ test, with values of *P* < 0.05 considered statistically significant.

## Results

### Search result

**Fig. 1:**
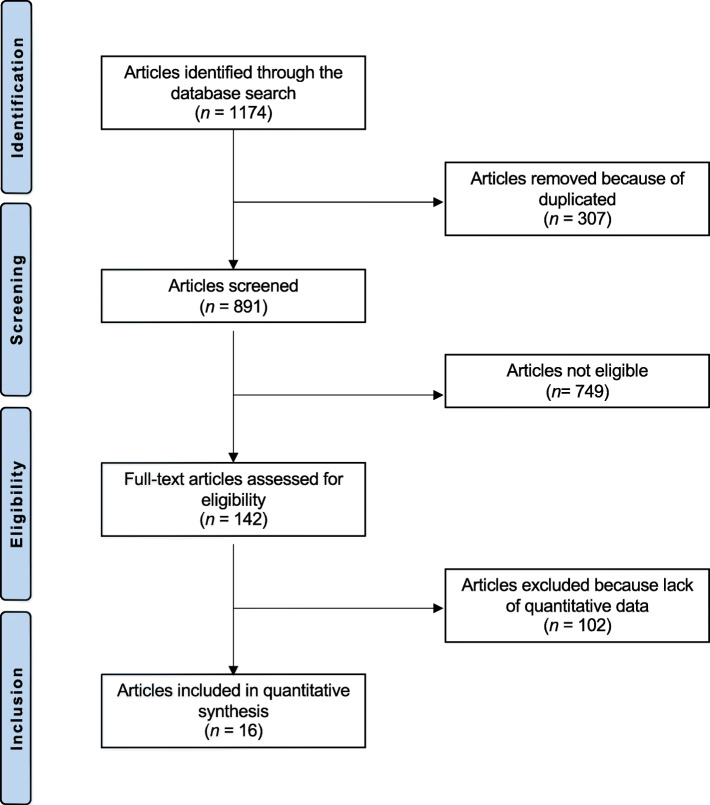
Flow chart of the literature search

The literature search resulted in 1174 studies. Of them, 307 were duplicates. A further 749 articles were excluded because of nature of the study (*N* = 233), non-clinical studies (*N* = 301), secondary osteoporosis (*N* = 81), small population or short follow-up (*N* = 19), multiple therapies (*N* = 21), language limitations (*N* = 9), uncertain results (*N* = 13), and others (*N* = 72). Another 102 articles were excluded because data under the outcomes of interest were missing. Finally, 16 RCTs were eligible for inclusion in the present study (Fig. 1).

### Methodological quality assessment

**Fig. 2:**
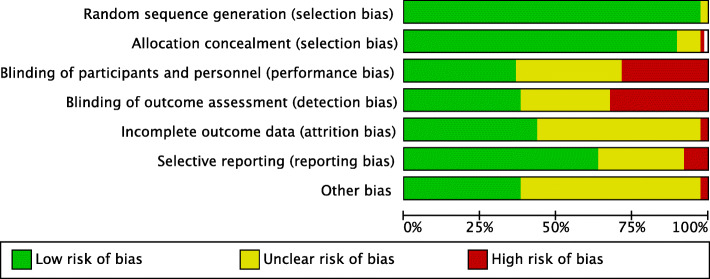
Methodological quality assessment

The inclusion of only RCTs yields to low risk of selection bias. Many studies performed patients and assessor blinding, thus leading to moderate-low risk of detection and performance biases. The overall high quality of the included studies led also to an overall low-risk of attrition and reporting bias. Overall, the results of the evaluation of each risk of bias item for each individual study included in the present analysis was low to moderate, leading to a good assessment of the methodology. The risk of bias graph is shown in Fig. 2.

### Patient demographics

A total of 14,446 patients were included. The median age was 67 (IQR 4.7), and the median BMI 25.4 (IQR 1.9). The median vertebral BMD was 0.82 (IQR 0.14), hip BMD 0.79 (IQR 0.1), and femur BMD 0.64 (IQR 0.02). The ANOVA test found optimal within-group variance concerning mean age, BMI, and BMD (*P* > 0.1). Generalities and patient baseline data of the included studies are shown in detail in Table 1.

**Table 1:** Generalities and patient baseline data of the included studies

Author, year	Journal	Mean follow-up (***months***)	Mean calcium daily supplement (***mg***)	Mean vit D daily supplement (***UI***)	Treatment	Administration	Samples (***n***)	Mean age	Mean BMI (***kg/m2***)	Mean BMD Spine (***g/cm***^***2***^)	Mean BMD Hip (***g/cm2***)	Mean BMD Femur neck (***g/cm***^***2***^***)***
Bone et al., 1997 [18]	*J Clin Endocrinol Metab*	24	813		Alendronate	OS	86	71				
			880		Alendronate	OS	89	70				
			831		Alendronate	OS	93	71				
			900		Placebo	OS	91	71				
Brown et al., 2014 [31]	*Osteoporos Int*	12			Denosumab	SC	852	68				
					Ibandronate	OS	851	67				
					Risedronate	OS						
Chung et al., 2009 [32]	*Calcif Tissue Int*	6	500	125	Ibandronate/Risedronate	OS	176	61	23.30			
					Risedronate/Ibandronate	OS	176	62	23.40			
					Placebo	OS	44	70	25.10	0.75		0.61
Delmas et al., 2002 [33]	*J Clin Endocrinol Metab*	48	500	400-600	Raloxifene	OS	2259	66	25.30	0.82		0.62
					Raloxifene	OS	2277	66	25.20	0.81		0.62
					Placebo	OS	2292	67	25.30	0.81		0.62
Gonnelli et al., 2014 [34]	*Bone*	12	841	400	Zoledronate	IV	30	66	26.10	0.82	0.79	
			870		Ibandronate	IV	30	67	25.70	0.82	0.79	
Grey et al., 2012 [35]	*J Clin Endocrinol Metab*	12	960		Zoledronate	IV	43	64		1.01	0.85	
			880		Zoledronate	IV	43	66		1.03	0.84	
			850		Zoledronate	IV	43	66		1.05	0.84	
			950		Placebo	IV	43	65		1.03	0.87	
Harris et al., 1999 [19]	*JAMA*	36	1000	500	Risedronate	OS	817	69	26.60	0.84		0.60
					Risedronate	OS	821	69	26.60	0.83		0.59
					Placebo	OS	820	68	26.50	0.83		0.60
Hooper et al., 2005 [36]	*Climacteric*	24			Risedronate	1OS	128	53		1.08		
					Risedronate	OS	129	53		1.08		
					Placebo	OD	126	53		1.08		
Iwamoto et al., 2008 [37]	*Yonsei Med J*	12	800		Alendronate	OS	61	70	21.90	0.62		
					ECT	OS	61	69	21.70	0.65		
Iwamoto et al., 2011 [38]	*Osteoporosis Int*	6	800		Alendronate	OS	97	78	22.00			
					Raloxifene	IM	97	82	21.90			
Leder et al., 2014 [39]	*J Clin Endocrinol Metab*	24			Teriparatide	SC	31	66	25.50	0.82		0.64
					Denosumab	SC	33	66	24.10	0.87		0.64
					Combined	SC	30	66	25.40	0.86		0.64
Liang et al., 2017 [40]	*Orthop Surg*	24			Zoledronate	IV	155	57	21.80	0.63	0.75	
					Placebo	IV	95	57	21.60	0.63	0.75	
Lufkin et al., 1998 [41]	*J Bone Min Res*	12			Raloxifene	OS	48	67	24.80	0.75	0.64	
					Raloxifene	OS	47	67	26.20	0.81	0.69	
			750	400	Calcium/Vit D	OS	48	68	25.30	0.77	0.67	
Muratore et al., 2010 [42]	*Adv Ther*	12	1000	800	Clodronate	IM	28	64				
					Clodronate	IM	32	64				
Paggiosi et al., 2014 [43]	*Osteoporos Int*	24	1200	800	Alendronate	OS	57	68	25.90	0.79	0.75	0.64
					Ibandronate	OS	58	67	26.40	0.80	0.78	0.64
					Risedronate	OS	57	67	26.80	0.81	0.80	0.67
					Control		226	38	25.10	1.,07	0.97	0.86
Roux et al., 2014 [44]	*Bone*	12	≥1000	≥800	Denosumab	SC	435	68				
					Risedronate	OS	435	68				

### Outcomes of interest

**Fig. 3:**
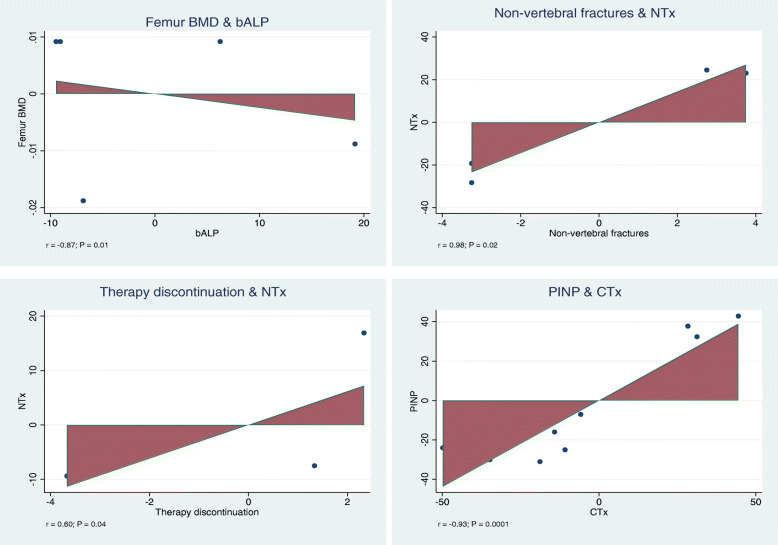
Added variable plots of the statistically significant outcomes

Greater bALP was associated with lower femoral BMD (*r* = − 0.87; *P* = 0.01). Greater NTx was associated with greater of occurrence of non-vertebral fractures (*r* = 0.98; *P* = 0.02). Greater NTx was associated with greater rate of therapy discontinuation (*r* = − 0.60; *P* = 0.04). There was evidence of positive association between PINP and CTx (*r* = −0.93; *P* = 0.0001). No other statistically significant associations were detected. These results are shown in Table 2. Added-variable plots of the statistically significant outcomes are displayed in Fig. 3.

**Table 2:** Overall results of the multivariate analysis

Endpoint	bALP		NTx		bCTx		PINP	
	***P***	***r***	***P***	***r***	***P***	***r***	***P***	***r***
BMD spine	0.6	0.16	0.06	−0.59	0.9	0.0	1.0	0.00
BMD hip	0.5	0.40	0.3	−0.83	0.9	0.0	0.8	−0.13
BMD femur	*0.01*	*−0.87*	0.4	−0.29	0.6	−0.3	1.0	−1.00
Non-vertebral fractures	0.2	−0.41	*0.02*	*0.98*	1.0	−1.0		
Vertebral fractures	0.6	−0.18	0.5	0.29				
Femur fractures					1.0	−1.0		
Hip fractures					1.0	−1.0		
Adverse events	0.08	−0.65	0.5	−0.44	0.9	0.0	0.3	−0.58
Serious adverse events			0.5	0.68	0.7	−0.3		
Therapy discontinuation	0.2	−0.46	*0.04*	−*0.60*	0.4	−0.4	0.5	−0.55
Gastrointestinal adverse events	0.5	−0.40	0.8	−0.25	0.2	−0.9	0.2	−0.97
Musculoskeletal adverse events	0.1	−0.99			0.9	0.0	0.8	−0.15
Mortality					1.00	−1.0

## Discussion

Our findings suggest that bALP and NTx may represent useful, valid, and reliable tools for therapy monitoring for postmenopausal osteoporotic patients. Higher bALP and NTx were associated to lower femoral BMD and higher rate of non-vertebral fractures, respectively. Furthermore, a positive association between NTx and the rate of adverse events leading to therapy discontinuation was evidenced. BTMs are implicated in bone turnover, and their level significantly varies during osteoporotic therapy [31, 33, 37, 38, 45]. P1NP and bCTx did not show any statistically significant association with any of the considered variables in the present study. PINP is released following to the amino/carboxy-terminal extensions cleavage of the procollagen and can be found variably in the blood [46]. bCTx is a form of the telopeptides of type I collagen released during collagen degradation [7, 47]. We were unable to find any significant association for these two BTMs; thus, their potential in therapy monitoring remains uncertain.

Given their sensibility to reveal changes in bone turnover, BTMs gained popularity [3, 22, 23]. BTMs’ variations related also to antiresorptive drugs, which produce a quick decrease of the bone resorption markers, followed by those of bone formation [48]. Vice versa, anabolic drugs increase the level of bone formation markers, followed by those of bone resorption [45]. BTMs’ changes are related to the risk of fragility fractures; thus, BTMs have been introduced to monitor therapy in osteoporotic patients [49, 50]. The effect of the therapy on BTMs strictly depends on the type of drug used [45]. Antiresorptive drugs inhibit osteoclasts and cause a rapid reduction in resorption markers, followed by a reduction in bone formation markers [45]. Indeed, the present study shows an association between BMD and bALP. BALP is a membrane-bound enzyme found in almost all tissues of the organism and can be easily measured in serum [51]. bALP has been the first BTMs of bone turnover intensively investigated [52]. It was initially used to monitor the efficacy and safety of some drugs acting on bone turnover, and subsequently acquired popularity to monitor therapy in osteoporotic patients [53, 54]. Bjarnason et al. [55] found that bALP had stronger association than BMD to predict the risk of fragility fractures in patients undergoing raloxifene therapy. These results were unexpected, since BMD was considered a very reliable measure of the risk of fractures [56, 57]. Similar results were found by Iwamoto et al. [38] and Gonnelli et al. [34] evaluating the outcome of alendronate zoledronate and ibandronate on patients’ quality of life. Both studies evidenced a statistically significant association between the increase in BMD and a decrease in serum bALP levels. Comparable results were obtained by Delmas et al. [33], evaluating the efficacy of raloxifene in preventing vertebral fracture in patients with postmenopausal osteoporosis. Muratore et al. [42] found that the bALP and BMD variations were proportional to the dose of clodronate administered to patients. Overall, these findings encouraged the use of bALP to monitor therapy in patients undergoing pharmacological management of postmenopausal osteoporosis [42].

Our analyses showed evidence of positive association between NTx and the rate of non-vertebral fractures. NTx and bCTX are two different forms of the telopeptide of type I collagen, which modulate the degradation process of collagen [7, 47]. These telopeptides are measurable in serum and in the urines and exhibit to circadian cycle variations [58, 59]. Iwamoto et al. [38] demonstrated that alendronate reduced the urinary level of NTx [38]. Garnero et al. [60] found that the urinary excretion of NTx did not predict fractures, hypothesizing that it follows a different pattern of bone collagen degradation.

This study shows limitations. The analyses were performed regardless to the drug type and administration. This enhanced the risk of bias of the present study, negatively affecting the reliability of our results. Furthermore, the heterogeneous daily administration of vitamin D and calcium represents another important limitation. We included only RCTs reporting quantitative data under the outcomes of interest, which were published in peer reviewed scientific. However, the role of BTMs has been poorly investigated, and none of the included articles did not aim to quantify directly the biomarkers’ variations. Results from this study should encourage future investigation to evaluate the potential of BTMs in a clinical setting, analyzing their variations as primary outcome. The biological variability of BTMs constitutes an important factor limiting their engagement in the management of osteoporosis.

## Conclusion

The analysis of BTMs in the investigation of their possible role in monitoring therapy demonstrates the need for studies that can validate their use in clinical practice. Our analysis supports the adoption of BTMs in therapy monitoring of postmenopausal osteoporosis patients. Further studies are needed to analyze variations of BTMs in relation to treatment as a primary outcome.

## Data Availability

This study does not contain any third material.

## Abbreviations

BTMs: Biochemical markers of bone turnover
bALP: Bone alkaline phosphatase
PINP: Procollagen type I N propeptide
bCTx: Serum cross-linked C-telopeptides of type I collagen
NTx: Urinary cross-linked N-telopeptides of type I collagen
RCTs: Randomized clinical trials
BMI: Bone mass index
BMD: Bone mineral density
